# Antibacterial and antifungal drug concentrations in intra-abdominal abscesses: a prospective clinical study

**DOI:** 10.1128/aac.01178-24

**Published:** 2024-12-05

**Authors:** Alicia Cancela Costa, Fabian Grass, Ignacio Andres Cano, Florian Desgranges, Constant Delabays, Antonios Kritikos, Emmanouil Glampedakis, Thierry Buclin, Rafael Duran, Benoit Guery, Jean-Luc Pagani, Emilie Uldry, Laurent Arthur Decosterd, Frederic Lamoth

**Affiliations:** 1 Service of Infectious Diseases, Department of Medicine, Lausanne University Hospital and University of Lausanne 30635 , Lausanne, Switzerland; 2 Department of Visceral Surgery, Lausanne University Hospital and University of Lausanne 30635 , Lausanne, Switzerland; 3 Department of Radiology and Interventional Radiology, Lausanne University Hospital and University of Lausanne 30635 , Lausanne, Switzerland; 4 Service and Laboratory of Clinical Pharmacology, Lausanne University Hospital and University of Lausanne 30635 , Lausanne, Switzerland; 5 Adult Intensive Care Service, Lausanne University Hospital and University of Lausanne 30635 , Lausanne, Switzerland; 6 Institute of Microbiology, Department of Laboratory Medicine and Pathology, Lausanne University Hospital and University of Lausanne 30635 , Lausanne, Switzerland; University Children's Hospital Münster, Münster, Germany

**Keywords:** piperacillin, meropenem, imipenem, ertapenem, fluconazole, caspofungin, anidulafungin, bioavailability, peritonitis, surgical

## Abstract

Secondary peritonitis with intra-abdominal abscesses (IAA) is difficult to treat because of the supposed low rate of penetration of antimicrobial drugs at the site of infection. However, clinical data about the actual bioavailability of antimicrobial drugs in IAA are scarce. This prospective observational study aimed at assessing the drug penetration in IAA of the antibiotics (piperacillin-tazobactam, carbapenems) and antifungals (fluconazole, echinocandins) that are usually recommended for the treatment of intra-abdominal infections. Patients with IAA who underwent a radiological or surgical drainage procedure were included. Antimicrobial drug concentrations were measured in IAA (C_IAA_) and in a simultaneous plasma sample (C_plasma_) to assess the C_IAA_/C_plasma_ ratio. The pharmacodynamic target was defined as a C_IAA_ equal or superior to the clinical breakpoints of susceptibility of the most relevant intra-abdominal pathogens. Clinical outcomes were assessed at hospital discharge. A total of 54 antimicrobial drug measurements were performed in 39 IAA samples originating from 36 patients. Despite important inter-individual variability, piperacillin-tazobactam exhibited the highest C_IAA_/C_plasma_ ratios (median 2). The rates of target achievement were 75%–80% for piperacillin-tazobactam and meropenem but 0% for imipenem and ertapenem. These results tended to correlate with clinical outcomes (96% success rate versus 73%, respectively, *P* = 0.07). Among antifungals, fluconazole exhibited higher C_IAA_/C_plasma_ ratios and rates of target achievement compared to echinocandins. However, no differences in clinical outcomes were observed. These results provide unique information about antimicrobial drug penetration in IAA in real clinical conditions and suggest that piperacillin-tazobactam and meropenem may have better efficacy compared to imipenem or ertapenem.

## INTRODUCTION

Secondary peritonitis and intra-abdominal abscesses (IAA) are frequent complications of abdominal infections or surgery (1, 2). These infections represent a major cause of sepsis and are associated with high mortality rates (10%–30%) (1, 2). Both bacteria (mainly Enterobacterales and anaerobes) and fungi (*Candida* spp.) can be involved, which requires the use of broad-spectrum antibacterial drugs (carbapenems, piperacillin-tazobactam) or antifungal drugs (echinocandins, triazoles) (1–3). Furthermore, source control (peritoneal lavage and/or drainage of IAA) is crucial for outcome but cannot always be completely achieved (3). Persistence of voluminous IAA is associated with worse outcome in the absence of drainage (4). The limited penetration of antimicrobial drugs within IAA may contribute to the failure of therapy. While the actual distribution of antibiotics in IAA is supposed to be low, few studies have addressed this question. Most data are derived from animal models (5–10), and analyses in human clinical samples are limited to case reports or case series (9, 11–14). These studies are relatively old, and data about the broad-spectrum beta-lactams that are currently in use (e.g., piperacillin-tazobactam, carbapenems) are scarce. Regarding antifungal agents, data are also limited. Zhao et al. reported a better distribution of rezafungin compared to micafungin in a murine model of intra-abdominal candidiasis (15). Data about azole or echinocandin concentrations in peritoneal cavity, IAA, or other abscesses (e.g., soft tissues, brain) have been reported in a few case series or case reports (14, 16–18).

The aim of the present study was to compare IAA and plasma concentrations of the broad-spectrum antibacterial and antifungal drugs that are currently recommended for the treatment of complicated intra-abdominal infections and to correlate these results with clinical outcomes.

## RESULTS

### Characteristics of patients and microbiological samples

A total of 36 adult patients with IAA who underwent a drainage procedure were included. The characteristics of patients, intra-abdominal infections, and antimicrobial therapy including drug dosages are provided in Table 1. A total of 39 pus samples from 31 peritoneal and 8 liver abscesses were collected during a surgical or radiological procedure (*n* = 21) or via an already inserted drain (*n* = 18). Cultures of these 39 samples grew only bacteria in 18 (46%) cases, only fungi in 6 (15%) cases, and a mix of bacteria and fungi in 9 (23%) cases, while no pathogens could be isolated in the remaining 6 (15%) cases. The majority of bacterial pathogens recovered in culture consisted of *Escherichia coli* or other enterobacteria (62%). Fungi were all *Candida* spp. with a predominance of *Candida albicans* (71%). The volumes of the abscesses were measured, and there was no statistical difference between the different antimicrobial treatment groups (*P* = 0.39, Table 2). Antimicrobial therapy courses (*n* = 38) consisted of antibacterial monotherapy (piperacillin-tazobactam or a carbapenem), antifungal monotherapy (fluconazole or an echinocandin), and combined antibacterial and antifungal therapy in 20 (53%), 3 (8%), and 15 (39%) cases, respectively (Table 1).

**TABLE 1:** Characteristics of the patients and intra-abdominal (IA) infections^*i*^

Parameter	Value
Demographic characteristics (*n* = 36)	
Age (years)	69 (30–91)
Men/women	23 (64)/13 (36)
Underlying causes of IA abscesses (*n* = 36)	
Postoperative leakage/perforation	14 (39)
Diverticulitis	5 (14)
Pancreatitis	5 (14)
Neoplasia	5 (14)
Cholecystitis	2 (6)
Bowel inflammatory disease	2 (6)
Other	3 (8)
Localization of IA abscesses (*n* = 39)	
Peritoneal	31 (79)
Hepatic	8 (21)
Microbial pathogens in IA abscesses (*n* = 70)	
*Escherichia coli*	15 (21)
Other Enterobacterales	18 (26)
Non-fermentative Gram-negative bacteria	5 (7)
Gram-positive bacteria	14 (20)
Strict anaerobes	1 (1)
*Candida albicans*	12 (17)
Non-*albicans Candida* spp.	5 (7)
Antimicrobial therapy (*n* = 38)^*a*^^*,^j^*^	
Piperacillin-tazobactam^*b*^	12 (32)
Meropenem^*c*^	2 (5)
Imipenem^*d*^	5 (13)
Ertapenem^*e*^	1 (3)
Fluconazole^*f*^	2 (5)
Caspofungin^*g*^	1 (3)
Piperacillin-tazobactam^*b*^ and caspofungin^*g*^	3 (8)
Piperacillin-tazobactam^*b*^ and anidulafungin^*h*^	1 (3)
Piperacillin-tazobactam^*b*^ and fluconazole^*f*^	3 (8)
Meropenem^*c*^ and caspofungin^*g*^	3 (8)
Imipenem^*d*^ and caspofungin^*g*^	2 (5)
Imipenem^*d*^ and anidulafungin^*h*^	1 (3)
Ertapenem^*e*^ and anidulafungin^*h*^	1 (3)
Ertapenem^*e*^ and fluconazole^*f*^	1 (3)

^*a*^ Two patients underwent two courses of different antimicrobial therapies.

^*b*^ Piperacillin-tazobactam dosage was 4,500 mg q8h (*n* = 16) and 4,500 mg q6h (*n* = 3).

^*c*^ Meropenem dosage was 1,000 mg q8h (*n* = 3) and 2,000 mg q8h (*n* = 2).

^*d*^ Imipenem dosage was 500 mg q6h (*n* = 2), 750 mg q6h (*n* = 3), and 1,000 mg q6h (*n* = 3).

^*e*^ Ertapenem dosage was 1,000 mg q24h (*n* = 3).

^*f*^ Fluconazole dosage was 400 mg q24h (*n* = 5) and 400 mg q12h (*n* = 1).

^*g*^ Caspofungin dosage was 70 mg (loading dose) and then 50 mg q24h (*n* = 9).

^*h*^ Anidulafungin dosage was 200 mg (loading dose) and then 100 mg q24h (*n* = 3).

^*i*^ Numbers are median (range) for continuous variables and number (percentage) for proportions.

^*j*^ Dosing adjustment (according to an internal protocol) was performed in nine episodes (23%) because of altered renal function (glomerular filtration rate <70 mL/min). For these cases, the dosage mentioned in the table has been extrapolated to the recommended dosing for normal renal function.

**TABLE 2:** Antimicrobial drug concentrations in intra-abdominal abscesses and plasma samples^*k*^

Antimicrobial drug	Abscess volume [cm^3^]^*a*^	Day of therapy [d]^*a*^^,*b*^	Time from last dose [h]^*a*^^,*b*^	C_IAA_ [µg/mL]^*a*^	C_plasma_ [µg/mL]^*a*^	C_trough_ [µg/mL]^*a*^	Ratio C_IAA_/C_plasma_^*a*^	Target achievement^*c*^,^*d*^	Attributable failure^*c*^^,*e*^
Piperacillin/tazobactam^*f*^^,^^*g*^ (*n* = 20)	125 (12.7–2175)	9 (3–23)	4.8 (1.5–9.25)	21.6 (0–247.4) 3.4 (0.5–14.0)	8.3 (0.6–206) 1.4 (0.2–28.5)	7.5 (0.6–70.4) 0.9 (0.2–9.0)	2.06 (0–44.2) 2.39 (0.07–26)	15^*h*^ (75)	1 (5)
Meropenem (*n* = 5)	217.7 (27.2–427.8)	7 (2–20)	7.8 (6.25–8.75)	2.9 (0.3–5.7)	7.5 (0.2–20.3)	6.6 (0.5–8)	0.49 (0.04–14.5)	4 (80)	0 (0)
Imipenem/cilastatin^*i*^ (*n* = 8)	73.5 (22–377.8)	10.5 (2–23)	5.9 (2.75–7.5)	0.1 (0–0.12) 3.81 (0–19.1)	5.1 (0.9–22.1) 4.95 (1–33.2)	4.7 (0.9–22.1) 6.2 (2.1–33.2)	0.02 (0–0.04) 0.68 (0–1.98)	0^*j*^ (0)	2 (25)
Ertapenem (*n* = 3)	274.2 (11.1–348.5)	28 (10–77)	17.5 (17–22)	0.3 (0.1–0.3)	0.8 (0.1–5.7)	0.8 (0.1–3.7)	0.38 (0.05–1)	0 (0)	1 (33)
Fluconazole (*n* = 6)	208.8 (82.2–2175)	6 (2–63)	17.4 (2.5–24)	12.7 (5–24.5)	18.1 (5.7–27.1)	15.2 (5.7–27.1)	0.85 (0.54–1.1)	6 (100)	0 (0)
Caspofungin (*n* = 9)	245.6 (25.1–826.9)	11 (2–21)	13.5 (1.5–23.75)	0.3 (0–1.6)	2.4 (1.2–3.6)	1.8 (1.2–2.9)	0.11 (0–0.75)	5 (56)	2 (22)
Anidulafungin (*n* = 3)	182.3 (11.1–293.2)	5 (5–18)	16 (12–22)	0 (0–0.2)	2.7 (2.4–3.3)	2.7 (1.5–3.3)	0 (0–0.07)	0 (0)	0 (0)

^*a*^ Results are median (range).

^*b*^ At time of drainage procedure.

^*c*^ Results are total number of cases (percentage).

^*d*^ Target achievement defined as C_IAA_ ≥ clinical breakpoints (CBP) using the CLSI CBPs for Enterobacterales (piperacillin 8 µg/mL, meropenem 1 µg/mL, imipenem 1 µg/mL, ertapenem 0.5 µg/mL) and *Candida albicans* (fluconazole 2 µg/mL, caspofungin 0.25 µg/mL, anidulafungin 0.25 µg/mL) (19, 20).

^*e*^ Attributable failure was defined as lack of target achievement (insufficient C_IAA_) and recovery of a microorganism that was susceptible to the ongoing antimicrobial therapy in a subsequent drainage intervention.

^*f*^ Tazobactam measurements were performed for 17 samples.

^*g*^ Results are displayed for both the piperacillin component (upper row) and the tazobactam component (lower row).

^*h*^ The target achievement was based on concentration of the active component piperacillin.

^*i*^ Results are displayed for both the imipenem component (upper row) and the cilastatin component (lower row).

^*j*^ The target achievement was based on concentration of the active component imipenem.

^*k*^ CIAA, concentration in intra-abdominal abscess; Cplasma, concentration in concomitant plasma sample; Ctrough, trough concentration measured before the next dose.

### Antimicrobial drug concentrations

**Fig 1:**
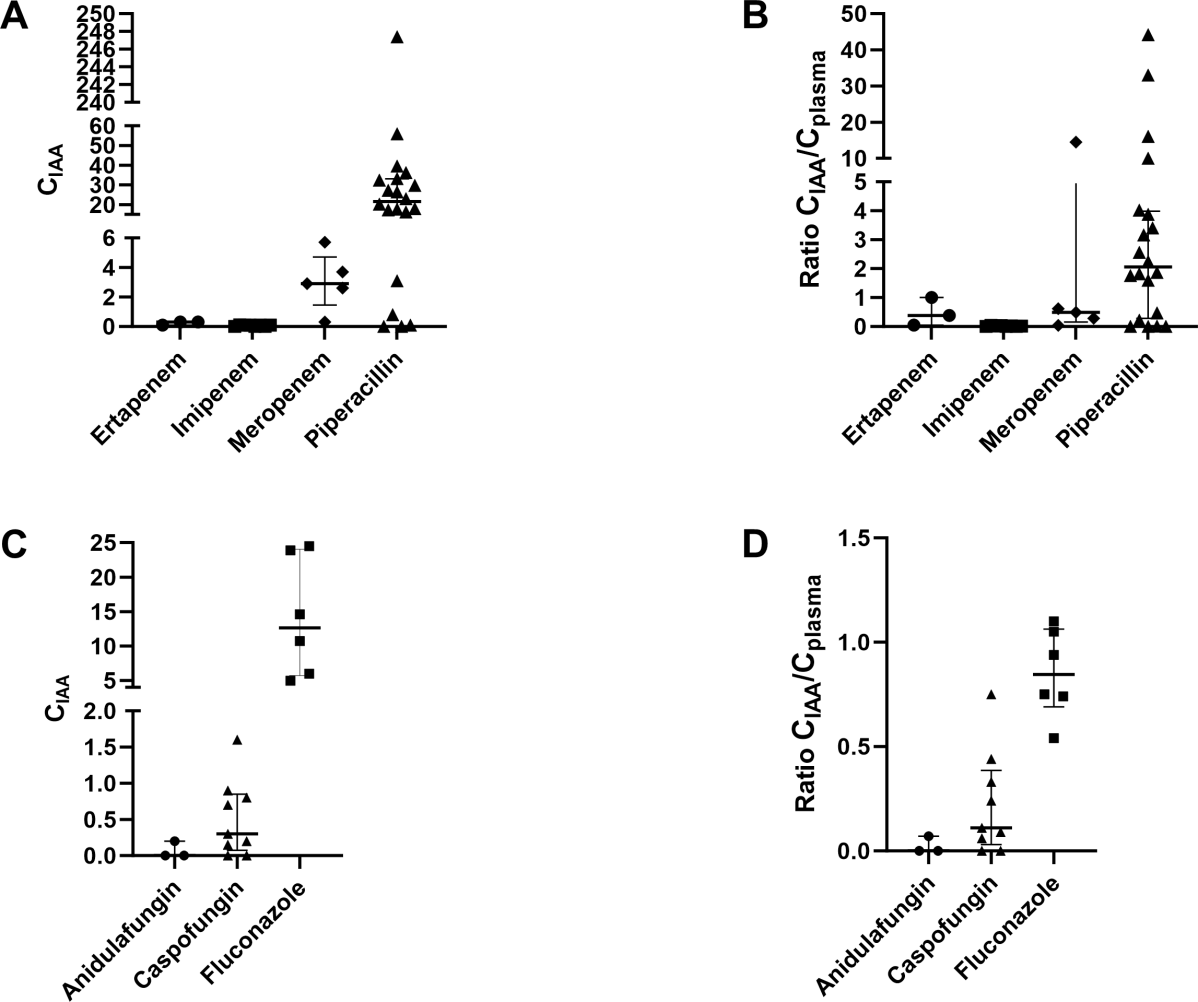
Antimicrobial drug penetration in intra-abdominal abscesses. The distribution of antimicrobial concentrations in the intra-abdominal abscesses (C_IAA_ [µg/mL]) and the ratio of the C_IAA_ and the concomitant concentrations in plasma (C_plasma_) is represented for each antibacterial drug (panels A and B) and each antifungal drug (panels C and D). Bars represent standard deviations.

Drug concentration measurements were performed in IAA samples (C_IAA_) and in concomitant plasma samples (C_plasma_) for meropenem (*n* = 5), imipenem-cilastatin (*n* = 8), ertapenem (*n* = 3), piperacillin-tazobactam (*n* = 20), fluconazole (*n* = 6), caspofungin (*n* = 9), and anidulafungin (*n* = 3) at a median of 9 days (range 2–77 days) after the start of antimicrobial therapy. Results of C_IAA_, C_plasma_, and C_IAA_/C_plasma_ ratios are shown in Table 2; Fig. 1. Overall, important inter-individual variability was observed for most antimicrobial agents. Among antibacterial drugs, piperacillin-tazobactam achieved the highest IAA penetration with median C_IAA_/C_plasma_ ratios > 2 for both components (piperacillin and tazobactam). All carbapenems displayed median ratios < 0.5, with meropenem and imipenem exhibiting the highest and the lowest values, respectively (0.49 vs 0.02, respectively *P* < 0.01). Among antifungal drugs, fluconazole displayed a significantly higher median C_IAA_/C_plasma_ ratio when compared to the echinocandin drugs caspofungin and anidulafungin (0.85 vs 0.08, respectively, *P* < 0.01).

A potential correlation between the abscess volumes and the C_IAA_/C_plasma_ ratios was analyzed for the drug with the highest sample size (piperacillin-tazobactam, *n* = 20); the result was not significant (Spearman’s coefficient: *r_s_* −0.01, *P* = 0.95). This analysis was not performed for other drugs because of the small sample size.

Trough plasma concentrations of the antimicrobial drugs (C_trough_) were also measured just before the administration of the next dose following the interventional procedure of drainage. Overall, median C_trough_ was close to median C_plasma_, suggesting modest interdose variability of plasma concentrations (Table 2).

### Pharmacodynamic analysis

To assess the appropriateness of IAA concentrations, we defined a pharmacodynamic target for each antimicrobial drug, which corresponded to the concentration to be achieved to overcome the clinical breakpoints (CBP) of the most relevant pathogens of intra-abdominal infections (see details in Materials and Methods). This target was achieved in the majority of cases (75%–80%) for piperacillin-tazobactam and meropenem but in none of cases (0%) for imipenem and ertapenem (Table 2). Among carbapenems, the difference in target achievement rate for meropenem when compared to imipenem and ertapenem was statistically significant (*P* < 0.01). For antifungals, target achievement rates were 100%, 56%, and 0% for fluconazole, caspofungin, and anidulafungin, respectively (Table 2). The difference between fluconazole and both echinocandins taken together was statistically significant (*P* = 0.04).

### Correlation with clinical outcomes

Failure of therapy occurred in 6/36 (17%) patients (i.e., five re-interventions of drainage and one death). Four episodes of failure occurred in patients for which the pharmacodynamic target was not achieved (i.e., C_IAA_ < CBP). In all four cases, microorganisms that were susceptible to the ongoing antimicrobial agent were recovered in the sampling of re-intervention, suggesting that underdosing may have been the cause of failure (Table 3). These cases of failure were attributed to imipenem (*n* = 2), ertapenem (*n* = 1), and piperacillin-tazobactam (*n* = 1). Two of these patients received concomitant caspofungin therapy, which was also considered as failure because of insufficient C_IAA_ and persistent positive *Candida* cultures in subsequent drainage samples (Table 3). Two patients experienced failure while receiving fluconazole therapy with C_IAA_ within the target. In these two cases, bacterial pathogens were recovered as the cause of failure, while fungal cultures were sterile, suggesting that fluconazole therapy was effective (Table 3). The rate of attributable failure per antimicrobial agent (i.e., insufficient C_IAA_ and susceptible pathogen recovered in subsequent drainage sample) is displayed in Table 2. For antibacterial agents, the rate of failure tended to be higher in patients receiving imipenem or ertapenem compared to those receiving piperacillin-tazobactam or meropenem (27% vs 4%, *P* = 0.07). For antifungal agents, there was no significant difference of failure rates between patients receiving fluconazole or an echinocandin.

**TABLE 3:** Analysis of cases of failure^*c*^

Case	Abscess localization and size [cm^3^]	Antimicrobial drug	Targeted microorganisms	C_IAA_ [µg/mL] (interpretation)^*a*^	Cause of failure, microorganisms recovered^*b*^
1	Peritoneal 826.9	Piperacillin-tazobactam	*E. coli*, *S. anginosus*, *S. mitis*	0.8 (not appropriate)	Recurrent intervention *E. coli*, *C. albicans*
		Caspofungin	*C. albicans*	0.2 (not appropriate)	
2	Hepatic 25.13	Imipenem	*K. pneumoniae*, *H. alvei*, *E. cloacae*, *E. faecalis*	0.1 (not appropriate)	Recurrent intervention *K. pneumoniae*, *H. alvei*, *C. albicans*
		Caspofungin	*C. albicans*	0 (not appropriate)	
3	Peritoneal 245.56	Ertapenem	*K. pneumoniae*, *C. freundii*, *S. anginosus*	0.3 (not appropriate)	Recurrent intervention *E. faecium*, *E. faecalis*, *L. rhamnosus*, *C. amalonaticus*, anaerobic flora
		Fluconazole	*C. albicans*	24.5 (appropriate)	
4	Peritoneal 21.97	Imipenem	*E. coli*, *K. pneumoniae*, anaerobic flora	0.1 (not appropriate)	Recurrent intervention *E. coli*, *K. pneumoniae*, *P. mirabilis*
5	Peritoneal 1964.94	Fluconazole	*C. albicans*	23.9 (appropriate)	Death (sepsis) *E. coli*
6	Peritoneal 138.53	Fluconazole	*C. albicans*	14.6 (appropriate)	Recurrent intervention *E. coli*, *E. faecium*

^*a*^ C_IAA_ was interpreted as appropriate if ≥8 µg/mL for piperacillin-tazobactam, 1 µg/mL for imipenem, 0.5 µg/mL for ertapenem, 0.25 µg/mL for caspofungin, and 2 µg/mL for fluconazole.

^*b*^ Failure was considered in case of persistent or recurrent collection requiring a re-intervention of drainage or death. Failure was attributed to insufficient antimicrobial drug concentration if a susceptible microorganism was recovered in the sampling of the re-intervention: case 1, *E. coli* (piperacillin-tazobactam) and *C. albicans* (caspofungin); case 2, *K. pneumoniae*, *H. alvei* (imipenem), and *C. albicans* (caspofungin); case 3, *C. amalonaticus* and anaerobic flora (ertapenem); case 4, *E. coli*, *K. pneumoniae*, and *P. mirabilis* (imipenem).

^*c*^ C_IAA_, antimicrobial concentration in intra-abdominal abscess.

## DISCUSSION

Human studies assessing antimicrobial drug penetration in abscesses are particularly scarce. Zimmermann et al. measured antimicrobial drug concentrations in 47 IAA of 42 patients without concomitant plasma levels (14). They assessed the adequacy of IAA concentrations using a similar approach compared to our study (i.e., C_IAA_ ≥ MIC). The proportion of adequate concentrations was acceptable for beta-lactams, such as piperacillin-tazobactam or cefepime (around 60%), and metronidazole (75%), but very low for ciprofloxacin, vancomycin, and fluconazole. Our results confirmed the relatively good penetration of piperacillin-tazobactam in IAA. Concentrations of both piperacillin and tazobactam in abscesses exceeded those in plasma in a majority of cases. However, it should be noted that tazobactam concentrations in both plasma and IAA were usually below the fixed concentration used for *in vitro* susceptibility testing (i.e., 4 µg/mL), although the significance of this observation is unknown. Overall, based on the piperacillin component, we estimated that IAA concentrations were adequate in 75% of cases.

Our results about carbapenems were quite different. While meropenem achieved acceptable C_IAA_/C_plasma_ ratios (median 0.5) and targeted IAA concentrations in 80% of cases, this was not the case for imipenem and ertapenem. The target was never achieved for these later drugs. Imipenem displayed particularly low C_IAA_/C_plasma_ ratios (<0.05 in all cases). Ertapenem exhibited better ratios (median 0.4), but concentrations were low in both IAA and plasma. Degradation of the drug, possibly due to inherent drug properties and/or the low pH conditions of abscesses, could be an explanation of these insufficient levels. Lack of stability of ertapenem has been previously reported (21). Imipenem is associated with cilastatin to prevent degradation by kidney enzymes (22). Whereas cilastatin also plays a role in preventing imipenem degradation in abscesses is unknown. Of note, we observed acceptable penetration of this compound in IAA (Table 2). Finally, inappropriate levels could also be attributed to insufficient dosage, notably for imipenem with dosage recommendations varying from 500 to 1000 mg q6h. However, we observed that all patients in our series exhibited very low C_IAA_, although they received the drug at different dosages including the highest one (Table 1).

Most interestingly, we found that these variable degrees of IAA penetration of broad-spectrum beta-lactam antibiotics tended to correlate with therapeutic response. Indeed, there was a trend towards better clinical outcomes for patients treated with piperacillin-tazobactam or meropenem compared to those treated with imipenem or ertapenem.

Regarding antifungals, our analysis showed that both drug penetration and rate of target achievement were significantly better for fluconazole compared to echinocandins. However, we did not find a difference for clinical outcomes. The fact that echinocandins have a larger spectrum and a better fungicidal activity against *Candida* spp. may compensate their lower IAA penetration compared to fluconazole and explain this absence of difference (23, 24).

Besides the small number size, there are some other limitations of the present study, which deserve mention. First, our results show important inter-individual variations of IAA concentrations and C_IAA_/C_plasma_ ratios, which may decrease the value of our results when considering their application for individual cases. However, these variations are in line with previous observations and may result from the large heterogeneity of the anatomical structure of IAA, including their localization, size, degree of inflammation, or fibrosis (9). Patients’ characteristics, including hemodynamic conditions, renal function, or albumin plasma levels, may also contribute to result heterogeneity. Contrarily to the results of a previous study (14), we did not observe a correlation between the size of the abscess and drug penetration in IAA.

Second, we defined the pharmacodynamic target as a C_IAA_ ≥ CBP. Although this approach is usually accepted for such analyses, it has not been validated and does not take into account important parameters that may affect the activity of antimicrobial agents, such as the protein binding. While the rates of protein binding of antimicrobials are known in plasma, data from other biological fluids, such as abscesses, are absent. It is expected that the biochemical conditions affecting protein binding (e.g., type of proteins present in the fluid and pH) may considerably differ between pus and plasma. Indeed, serum proteins that usually bind antimicrobial drugs (e.g., albumin, alpha-glycoprotein) are not supposed to diffuse in the interstitium in high amounts. Moreover, the impact of protein binding on antimicrobial activity is complex and debated (25). Despite this limitation, we found that our pharmacodynamic targets tended to correlate with clinical outcomes.

Third, although we found some correlation between antimicrobial drug bioavailability in IAA and clinical outcomes, this kind of analysis is hampered by multiple confounding factors, such as the number and size of abscesses, the type and efficacy of drainage procedure, the type and number of microorganisms recovered (e.g., bacterial, fungal, or mixed), and the use of combined antibacterial and antifungal therapy in some cases. Because all patients underwent a source control via a drainage procedure, we observed a relatively low rate of failure (17%), which limits the statistical power of such analysis.

Finally, there was a lack of uniformity regarding the timing of sampling, which was dependent on the timing of the drainage procedure. The IAA samples were collected at different time points within the interdose interval and sometimes relatively early after the start of antimicrobial therapy. However, we observed that the median C_trough_ were usually within the expected ranges.

In conclusion, our results provide unique information about the actual penetration of broad-spectrum beta-lactams and antifungals in IAA. Most importantly, piperacillin-tazobactam and meropenem achieved IAA concentrations that were deemed appropriate in most cases, which was not the case for imipenem and ertapenem. Despite some potential confounding factors, these results tended to correlate with clinical outcomes. However, these results should be interpreted cautiously because of the small sample size and would deserve further validation in larger data sets. The heterogeneity of data for a same drug also raises a warning about the generalization of these results for individual cases. While therapeutic drug monitoring for antimicrobial therapy usually relies on trough plasma level measurements, this parameter might not be appropriate for treatment of IAA considering the large variability of C_IAA_/C_plasma_ ratios.

## MATERIALS AND METHODS

### Study population

This prospective observational study was conducted at the Lausanne University Hospital (Switzerland) from September 2020 to January 2024. Adult (≥18-year-old) patients receiving broad-spectrum antibacterial drugs (piperacillin-tazobactam, meropenem, imipenem, ertapenem) or antifungal drugs (fluconazole, caspofungin, anidulafungin) for ≥48 h for the treatment of IAA and who underwent surgical or radiological drainage were eligible. Drugs were administered at recommended dosage (3, 26), which was adjusted to the estimated glomerular filtration rate (Cockroft-Gault formula) according to a standardized protocol established by the pharmacology service of our hospital.

The volumes of the abscesses were measured by two radiologists on the basis of the most recent abdominal computed tomography preceding the intervention. The following formula was used considering an ellipsoidal shape of most abscesses: length *x*-axis × *y*-axis × *z*-axis × 0.523. Patients were managed according to international recommendations for IAA treatment (3, 26, 27) at the discretion of the attending physicians and without intervention of the study investigators.

### Samples and measurements

Samples from the fluid content (pus) of the abscesses were collected for measurement of the drug concentration in IAA (C_IAA_) at the time of drainage or via an inserted drain. Plasma samples were collected concomitantly to the pus samples (± 1 h) and just before the next dose administration for measurement of C_plasma_ and C_trough_, respectively. Samples were stored at −80°C until analysis. Measurement of drug concentration was performed directly in a pus aliquot of 50 µL without pre-dilution by ultra-performance liquid chromatography-tandem mass spectrometry as previously described (28, 29). Drug penetration in IAA was assessed as the C_IAA_/C_plasma_ ratio.

### Pharmacodynamic and outcome analyses

A pharmacodynamic target was defined as a C_IAA_ that was equal or superior to the clinical breakpoints of susceptibility of the most frequent and relevant bacterial and fungal pathogens (i.e., *Enterobacterales* and *Candida* spp., respectively) according to the Clinical and Laboratory Standards Institute (CLSI) procedures (19, 20). These target thresholds were defined at 8 µg/mL for piperacillin-tazobactam (piperacillin component), 1 µg/mL for meropenem and imipenem, 0.5 µg/mL for ertapenem, 2 µg/mL for fluconazole, and 0.25 µg/mL for caspofungin and anidulafungin.

Outcomes were analyzed at hospital discharge. Failure of therapy was defined as a need of re-intervention for source control (surgical or radiological drainage) or death during hospital stay. The failure was attributed to the ongoing antimicrobial therapy in case of lack of target achievement (insufficient C_IAA_) and recovery of a microorganism that was susceptible to this antimicrobial drug in the subsequent drainage sample.

### Statistical analyses

The Fisher’s exact test was used for comparison of proportions and the Mann–Whitney or Kruskal–Wallis tests for comparison of two or multiple continuous variables, respectively. The correlation between two variables was assessed by Spearman’s rank correlation coefficient.
